# Identification of Genes Directly Involved in Shell Formation and Their Functions in Pearl Oyster, *Pinctada fucata*

**DOI:** 10.1371/journal.pone.0021860

**Published:** 2011-07-01

**Authors:** Dong Fang, Guangrui Xu, Yilin Hu, Cong Pan, Liping Xie, Rongqing Zhang

**Affiliations:** 1 Institute of Marine Biotechnology, School of Life Science, Tsinghua University, Beijing, China; 2 Protein Science Laboratory of the Ministry of Education, Tsinghua University, Beijing, China; University of Crete, Greece

## Abstract

Mollusk shell formation is a fascinating aspect of biomineralization research. Shell matrix proteins play crucial roles in the control of calcium carbonate crystallization during shell formation in the pearl oyster, *Pinctada fucata*. Characterization of biomineralization-related genes during larval development could enhance our understanding of shell formation. Genes involved in shell biomineralization were isolated by constructing three suppression subtractive hybridization (SSH) libraries that represented genes expressed at key points during larval shell formation. A total of 2,923 ESTs from these libraries were sequenced and gave 990 unigenes. Unigenes coding for secreted proteins and proteins with tandem-arranged repeat units were screened in the three SSH libraries. A set of sequences coding for genes involved in shell formation was obtained. RT-PCR and *in situ* hybridization assays were carried out on five genes to investigate their spatial expression in several tissues, especially the mantle tissue. They all showed a different expression pattern from known biomineralization-related genes. Inhibition of the five genes by RNA interference resulted in different defects of the nacreous layer, indicating that they all were involved in aragonite crystallization. Intriguingly, one gene (UD_Cluster94.seq.Singlet1) was restricted to the ‘aragonitic line’. The current data has yielded for the first time, to our knowledge, a suite of biomineralization-related genes active during the developmental stages of *P.fucata*, five of which were responsible for nacreous layer formation. This provides a useful starting point for isolating new genes involved in shell formation. The effects of genes on the formation of the ‘aragonitic line’, and other areas of the nacreous layer, suggests a different control mechanism for aragonite crystallization initiation from that of mature aragonite growth.

## Introduction

Biologically induced or controlled mineralization, also known as biomineralization, plays an essential role in a majority of metazoan taxa [1]. By depositing biogenic solid minerals, these processes construct a diversity of endo- and exo-skeletons [2]. The shells of mollusks are composed of calcium carbonate and small amounts of organic macromolecules (including proteins, polysaccharides and lipids) [3], and they provide one of the best known model systems for research on biomineralization [4]. Organic macro-molecules, especially proteins, account for less than 5% (w/w) of shell weight, but they are responsible for nucleation, polymorphism, orientation, morphology, and organization of calcium carbonate crystallites in the shell [5]. Several molluscan shell matrix proteins were separated and identified, and their distributions and functions in shell formation were extensively investigated in pioneering work on this topic [6], [7], [8], [9], [10], However, despite considerable efforts the detailed molecular mechanisms operating in shell biomineralization remain poorly understood. This is attributable to problems in protein separation and purification owing to a highly acidic amino acid content and protein complexation with minerals, as well as the absence of an effective experimental system [11].

The shell of the pearl oyster, *Pinctada fucata*, is comprised of inner aragonitic nacreous and outer calcitic prismatic layers. Matrix proteins have special effects on one or both layers. Nacrein [8], MSI60 [12], N16/pearlin [13], P10 [14], N19 [15], Pfi80 [16], and Pif97 [16] are involved in the nacreous layer, while MSI31 [12], MSI7 [17], prismalin-14 [18], aspein [19], KRMP family [20], prisilkin-39 [21], and the prismin family [22] are involved in the prismatic layer. In addition, the shematrin family [23] of proteins are involved in the formation of both nacreous and prismatic layers. One point worth mention is that, in the inner surface of the shell there is a section that connects the nacreous and prismatic layers, known as the ‘aragonitic line’, where a new biomineralizing conditions arise that result in a complete change in the mineralogy, shape, size, and growth modalities of biocrystals [24], [25], [26]. However, little is known about the formation of the sharp boundary between the calcitic and aragonitic domains. No proteins are yet known to control the structure of the ‘aragonitic line’.

Matrix proteins have several important characteristics. Firstly, they are characterized by the predominance of only a few amino acids, usually between two and four. Secondly, their primary structure is organized into different functional domains, including carbonic anhydrase domains and tandem-arranged repeat units [27]. Shell matrix proteins are secreted by the mantle tissue that covers the inner surface of the shell [28], [29], [30], [31], [32] and these proteins are likely to control crystal growth, so the mantle must have a key role in this process. The analysis of structure and expression pattern, together with *in vitro* crystallization, *in vivo* antigen injections [33], and the recently used RNAi approach [6], [16], are mainly used to investigate the function of the matrix proteins.

The developmental process of *Pinctada fucata* is classified into the following six stages: fertilized egg, trochophore, D-shaped stage, umbonal stage, juvenile, and adult [34], [35].The polymorphism of calcium carbonate crystals and the shell microstructure changes throughout these developmental stages. Prodissoconch I is formed during the early D-shaped stage. This first shell is considered to be composed of amorphous calcium carbonate (ACC) [36], a precursor of crystalline aragonite and calcite. The subsequent prodissoconch II is composed of aragonite with a homogeneous structure, while a thin prismatic structure appears in the late D-shaped and umbonal stages [31], [36]. Finally, the dissoconch shell with a prismatic layer and a nacreous layer is formed at the juvenile stage, which is the normal adult shell structure. Previous molluscan development research has focused on morphological changes in the shell. However, current knowledge about the changes at the gene expression level is very limited [30], [31], [34], [37] in the other species [38], [39], [40], [41]. The expression levels of six organic matrix genes during the ontogeny of *P. fucata* (nacrein, N16, MSI60, prismalin-14, aspein, and MSI31) were only recently investigated [36].

Research continues to build a connection between the expression of matrix proteins and the biomineralization of the *P. fucata* larval shell, so the discovery of new genes active at different developmental stages could lead to the identification of more biomineralization-related genes and enhance our understanding of molluscan shell formation. Therefore, we constructed three Suppression Subtractive Hybridization (SSH) libraries to isolate genes expressed at important developmental stages of shell formation. This study selected, sequenced, and annotated 2,923 positive clones with putative functions. These sequences were compared with known biomineralization-related genes and a small number returned a hit. A set of sequences coding for secreted proteins with tandem-arranged repeat units was identified. Thus, we recovered a subset of five uncharacterized genes that probably participate in shell bio-mineralization. The distribution of the five genes in several tissues was investigated, which showed that they were expressed by mantle cells with different expression patterns, thereby confirming our sequence predications. By using RNAi we found that knocking down these five genes gave rise to different disordered structures in the shell. All five genes were involved in the nacreous layer, and one (UD_Cluster94.seq.Singlet1) is the first gene known to participate in the formation of the ‘aragonitic line’. This study increases the known repertoire of biomineralization-related genes and highlights a new aspect of shell microstructure control.

## Results

### SSH library composition and characterization

**Figure 1:**
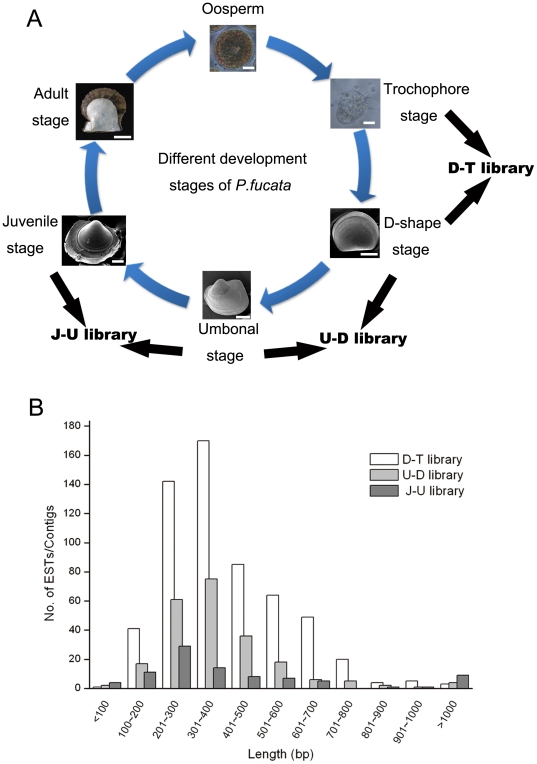
Overview of the three SSH libraries. (A) Schematic of SSH library construction. The D–T library was constructed using D-shape stage larval cDNA as the tester and trochophore stage larval cDNA as the driver. The U–D library was constructed using umbonal stage larval cDNA as the tester and D-shape stage larval cDNA as the driver. The J–U library was constructed using juvenile stage larval cDNA as the tester and umbonal stage larval cDNA as the driver. Bar  = 15 µm in oosperm, 15 µm in trochophore and D-shaped stages, 40 µm in umbonal and juvenile stages, and 4 cm in the adult stage. (B) Length distribution of unigenes in the three SSH libraries. The white columns represent unigenes in the D–T library, grey columns represent unigenes in the U–D library, and the dark grey columns represent unigenes in the J–U library. Unigene-length frequencies for each library are plotted in 100-bp bins.

Transcripts relevant to shell formation of *P.fucata* were isolated using an SSH procedure for the different development stages. Thus, three SSH libraries were constructed. The D–T library was constructed using cDNA prepared from the early D-shape stage (22-hour-old larvae) as the tester and that from trochophore stage (17-hour-old larvae) as the driver. The U–D library was obtained with cDNA prepared from the umbonal stage (7-day-old larvae) as the tester and that from D-shape stage (22-hour-old larvae) as the driver. The J–U library was obtained using cDNA from the juvenile stage (35-day-old larvae) and the umbonal stage (7-day-old larvae) as tester and driver, respectively (Figure 1A).

To evaluate the effectiveness of subtraction, we tested the reduction of GAPDH mRNA abundance in each library (Figure S1). These data showed that GAPDH mRNA abundance appeared to decrease by a factor of at least 1∶1024 in the subtracted library compared with the unsubtracted samples. Thus, we concluded that we successfully removed housekeeping genes in our three SSH libraries.

Differential cDNA fragments from the three libraries were screened using cDNA dot blots of the 3,515 clones (data not shown), *i.e*., we sequenced 990 positive sequences in the D–T library, 1,180 in the U–D library, and 753 in the J–U library. Quality trimming and clustering were performed and these sequences produced 2,376 contigs, which were assembled into 900 unigenes (see Table 1). In the D–T library, 484 sequences were singletons and library redundancy was 31%. In the U–D library, 177 sequences were singletons and library redundancy was 80%. In the J–U library, 70 sequences were singletons and library redundancy was 79%. The average cluster length was 396 bp in the D–T library, 376 bp in the U–D library, and 415 bp in the J–U library (Figure 1B).

**Table 1:** Summary of the three SSH libraries.

	Tested clones	Positive Clones	ESTs	Contigs	Unique clusters
D–T library	1,227	1,012	990	843	584
U–D library	1,257	1,196	1,180	1,130	227
J–U library	1,031	767	753	403	89
Total	3,515	2,975	2,923	2,376	900

### Gene ontology of SSH library unigenes

Unigenes were annotated by BLASTX searches against the GenBank nr database to identify unigenes with an e-value >10e–05, with the result that 29% were found in the D–T library, 38% in the U–D library, and 47% in the J–U library. Blast2GO [42] and WEGO [43] were used to annotate unigenes from the three libraries. Eighty-four (14%) D–T library unigenes, 52 (23%) U–D library unigenes, and 19 (21%) J–U library unigenes had hits in the gene ontology (GO) annotation and Uniprot databases. We found that the relative counts of subtracted genes were similar among the three libraries (figure S2). In the three libraries, the most highly represented classes for GO ‘molecular function’ were binding, catalytic activity, and transporter activity. The majority of genes with GO ‘biological process’ annotations were related to cellular processes and physiological processes. The ‘cellular location’ of the majority of genes in the three libraries was ‘cell’.

### Annotation of unknown unigenes

SSH procedures were performed in the development stages, so the sequences found in the libraries were involved in metamorphosis, growth, and shell formation. We isolated genes with different functions from these libraries.

In order to focus our analysis on genes involved in the biomineralization of *P.fucata*, we retrieved all unigenes with shared similarity using the GenBank nr database. Fifteen unigenes in the D–T library were similar to genes involved in biomineralization, *i.e*., chitin synthase [44], [45], [46], [47], chitin-binding protein [6], [48], [49], [50], collagen [51], [52], [53], [54], lectin [55], [56], [57], [58], [59], fibrinogen [60], [61], [62], and cysteine-rich protein [20]. Ten unigenes in U–T library returned a hit. They shared similarity with collagen, lectin, calmodulin, calcium/calmodulin-dependent protein kinase kinase [63], [64], [65], [66], [67], ferritin-like protein [68], [69], [70], [71], BMP [72], and matrix protein MSI7. In the J–U library, we found six unigenes using this approach. They shared high similarity with lectin, ferritein, matrix protein MSI7, and matrix protein MSI60 (Table S1). Interestingly, we found that DT_Cluster236 shared a similarity with the nicotinic acetylcholine receptor in *Takifugu rubripes.* In previous work, an ACC binding protein (ACCBP) was found to possess a nicotinic acetylcholine receptor [33]. Given the similar functional domain of DT_Cluster236, it might also be involved in the control of ACC in shell formation. Thus, we performed further *in situ* hybridization and RNAi analysis.

The mantle is the main tissue that controls shell formation, so genes expressed by mantle tissue would be more likely to be involved in shell biomineralization. We searched unigenes in the three SSH libraries against the transcriptome of the *P. fucata* mantle tissue (data not published) by BLASTN annotation. 446 unigenes returned a hit with a cutoff e-value of 10e–06.

### Secreted proteins

**Figure 2:**
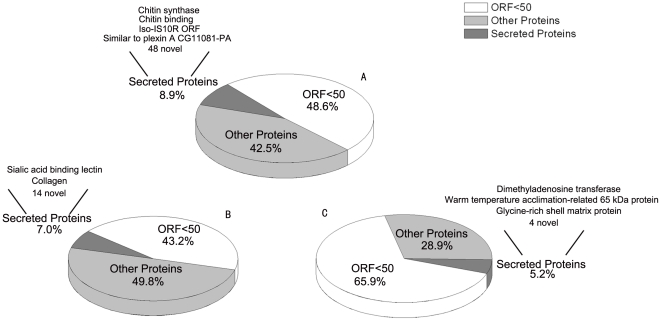
Secreted proteins predicted in the three SSH libraries. Unigenes with a coding region >150 base pairs, processing a signal sequence, predicted to be secreted proteins using TargetP [88] on TMHMM Server v. 2.0 (http://www.cbs.dtu.dk/services/TMHMM/), and GPI modification predictor [89] together [90]. The unigenes coding for secreted proteins were searched by BLASTX using the Genbank nr database with a cutoff e-value of 10e–05. (A) Unigenes in the D–T library. (B) Unigenes in the U–D library. (C) Unigenes in the J–U library.

Matrix proteins participate in the formation of the shell and these proteins are mainly secreted proteins. We retrieved conceptually derived full-length gene products that possessed a signal sequence, from the three substrate libraries. Based on the presence of conserved signal sequences and similarity to secreted proteins in GenBank (Figure 2), we found that 52 (8.9%) unigenes encoded secreted proteins in the D–T library, 16 (7.0%) in the U–D library, and 7 (5.2%) in the J–U library.

BLASTX analysis with a cutoff e-value of 10e-05 we found that most of the unigenes that coded for secreted protein in the three libraries, *i.e*., 88% in all, shared no similarity with sequences in the GenBank nr and EST databases. Some were similar to chitin-binding protein, collagen, sialic acid-binding lectin, and glycine-rich shell matrix protein (MSI7), which are known to be involved in biomineralization. As a control, we searched known matrix proteins in *P.fucata* and 80% of them encoded secreted proteins.

### Proteins with tandem-arranged repeat units

**Figure 3:**
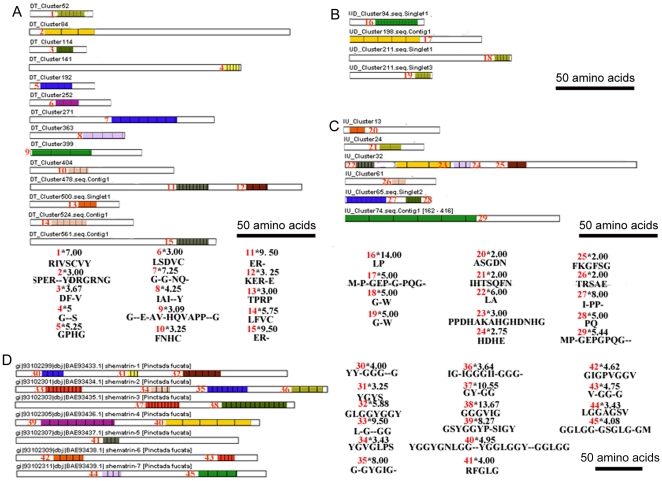
Schematic of unigenes coding for proteins with tandem-arranged repeat units. XSTREAM [73] was used to isolate unigenes coding for proteins with tandem-arranged repeat units. Unigenes in the D–T library (A), U–D library (B), and J–U library (C), with a coding region longer than 150 base pairs that were subjected to this analysis. As a control, shematrin family genes found in *P.fucata* were analyzed using XSTREAM (D). The identities of tandem-arranged repeat units are marked by numbers on the schematic at the top left of each motif (red font). The copy number of repeat units is shown on the top right of the motif (black font). On the top of each motif there is a label of identity*copy number. Bars  = 50 amino acids (in A, B, C, D).

An important feature of *P.fucata* biomineralization-related proteins is their modularity, which means that they are organized in different functional domains. Many of the domains are composed of tandem-arranged repeat units. We retrieved the known matrix proteins in *P.fucata*. XSTREAM [73] analysis found that 74.6% of the matrix proteins contained tandem-arranged repeat units. Data from the three libraries were then screened. We found fourteen unigenes from the D–T library, four unigenes from the U–D library, and six unigenes from the D–T library, which coded for proteins with tandem-arranged repeat units (Figure 3A–C).

As a control, we used shematrein family gene sequences as query sequences (Figure 3D). XSTREAM showed that all had tandem-arranged repeat units and most contained more than one. BLASTX search of the GenBank nr database was conducted with the 24 unigenes coding for proteins with tandem-arranged repeat units in our three SSH libraries. Only one (MU_Cluster65.seq.Singlet2) was a previously known *P. fucata* matrix protein MSI7, but seven unigenes coded for proteins similar to those found in other species, whereas the remainder shared no similarity. Analysis of secreted proteins found in the three libraries showed there were four secreted proteins with tandem-arranged repeat units, *i.e*., namely DT_Cluster252, DT_Cluster524.seq.Contig1, UD_Cluster94.seq.Singlet1, and IU_Cluster32.

### Spatial distribution of unigenes in mantle tissue

**Figure 4:**
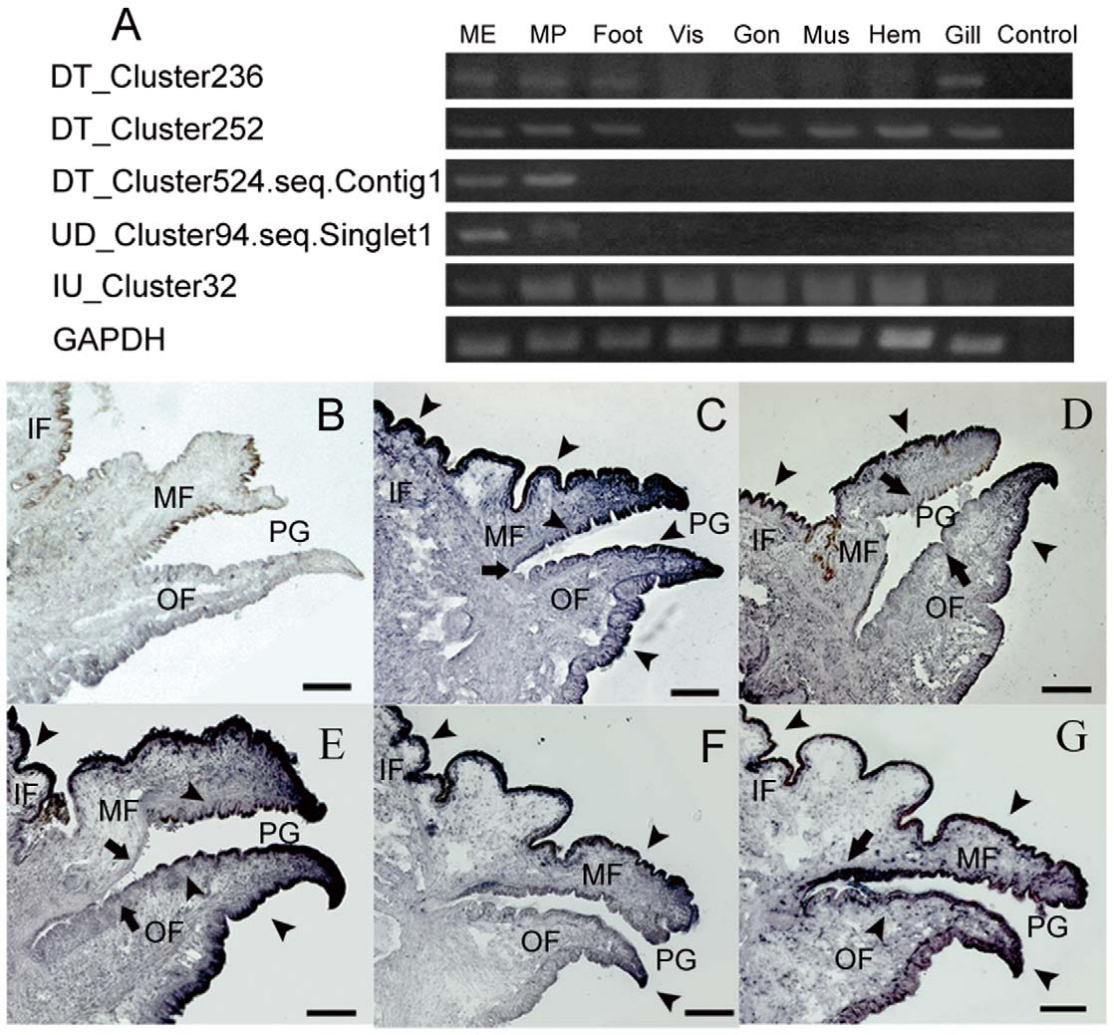
Gene expression patterns for selected genes in tissues of *P.fucata.* (A) Tissue-specific gene expression of selected unigenes by RT-PCR analysis. Total RNA was extracted from mantle edge (*ME*), mantle pallial (*MP*), foot, viscus (*Vis*), gonad (*Gon*), adductor muscle (*Mus*), hemocytes (*Hem*), and gill. RT-PCR was used without a template as a negative control (Control). The housekeeping gene GAPDH was used as a positive control. (B) A section of oyster mantle was hybridized with GFP anti-sense RNA probes as a negative control, and no hybridization signals (dark purple) were detected in this section. DT_Cluster236 (C) and DT_Cluster524.seq.Contig1 (E) were expressed in all mantle epithelial cells (indicated by arrowheads), except the cells at the bottom of the periostracal groove (indicated by arrows). (D) Expression of DT_cluster252 was localized to mantle epithelial cells (indicated by arrowheads) with weaker signals in the inner epithelial cells of the outer fold, and the outer epithelial cells of the middle fold at the periostracal groove (indicated by arrows). (F) Expression of UD_Cluster94.seq.Singlet1 was localized to the epithelial cells of the inner fold, the inner epithelial cells of the middle fold, and the epithelial cells at the top of the outer fold (indicated by arrowheads). (G) Expression of IU_Cluster32 was localized to nearly all the mantle epithelial cells (indicated by arrowheads), but at the bottom of the periostracal groove we detected stronger signals in epithelial cells (indicated by arrows). OF, outer fold; MF, middle fold; IF, inner fold; PG, periostracal groove. Scale bar, 0.5 mm.

Bioinformatics analysis isolated a set of unigenes that could be involved in shell formation. However, without a functional test their role in shell biomineralization was not clear and doubtful. To further characterize the unigenes that were directly involved in the shell formation, we further analyzed DT_Cluster236, which was predicted to be involved in the control of ACC, and the four unigenes that coded for secreted proteins with tandem-arranged repeat units. These five unigenes had a hit in BLASTN search against the transcriptome of mantle tissue (cutoff e-value 10e–06). By RT-PCR analysis, we tested the expressions of these five unigenes. Expression patterns of the five genes were different, *i.e*., DT_Cluster236 and IU_Cluster32 were expressed in all eight tissues; DT_Cluster252 was expressed in all eight tissues, except the viscus; whereas DT_Cluster524.seq.Contig1 and UD_Cluster94.seq.Singlet1 were only expressed in the mantle edge and mantle pallial (Figure 4A). Different shell types are formed by the ordered secretion of proteins and other molecules along the length of the mantle [2], so we further analyzed the distribution of these genes by *in situ* hybridization using frozen sections of the mantle. DT_Cluster236 and DT_Cluster524.seq.Contig1 were expressed in all the mantle epithelial cells, except the cells at the bottom of the periostracal groove (Figures 4C, 4E). *In situ* expression of the DT_cluster252 was localized to the mantle epithelial cells, with weaker signals in the inner epithelial cells of the outer fold, and the outer epithelial cells of the middle fold at the periostracal groove (Figure 4D). Expression of UD_Cluster94.seq.Singlet1 was localized to the epithelial cells of the inner fold, the inner epithelial cells of the middle fold, and the epithelial cells at the top of the outer fold (Figure 4 F). As with DT_Cluster236 and DT_Cluster524.seq.Contig1, the expression of IU_Cluster32 was localized to most of the mantle epithelial cells. However, we detected stronger signals in epithelial cells at the bottom of the periostracal groove (Figure 4G). Hybridization with a control GFP anti-sense RNA probe showed no significant signals (Figure 4B). Furthermore, it was noteworthy that the expression profiles were dissimilar to all known matrix proteins.

### RNAi knockdown of unigenes

By analysis the spatial distribution profiles of unigenes in mantle tissue, it is suggested that the mantle expressed five genes (DT_Cluster236, DT_Cluster252, DT_Cluster524.seq.Contig1, UD_Cluster94.seq.Singlet1, and IU_Cluster32) which were involved in shell formation. We then tested the functions of these five genes *in vivo* using RNAi. Controls were: KRMP, which is involved in the outer calcitic prismatic layer; nacrein, which is involved in the inner aragonitic nacreous layer; GFP, which is not expressed in *P.fucata*. Double strand RNA (dsRNA) was designed from the sequences of the eight genes and injected into the muscle of *P. fucata* (2 years old, shell length  = 5∼6 cm), and real-time quantitative PCR (RT-qPCR) measured expression levels of these eight genes six days after injection.

**Figure 5:**
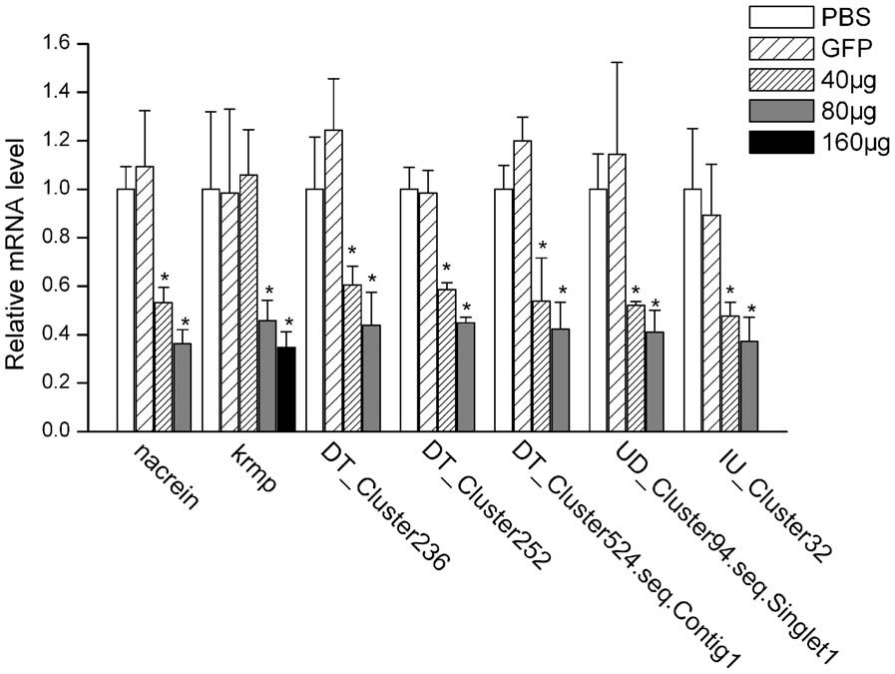
Expression levels of the selected genes knocked down by RNAi. The expression levels of the selected genes were measured using real-time quantitative PCR. The expression levels were analyzed six days after dsRNA injection and five individuals were tested in each group. For the control, the expression levels of the PBS injected group were measured to a relative value of 1.0. The asterisk represents a significant (*p*<0.005) difference compared with the PBS-injected groups. A 160 µg dosage was only used in the *krmp*-injected group, because the expression did not decrease significantly in the 40 µg dosage-injected group.

Expression levels of the 40 µg-injected groups were suppressed by approximately 50∼60% compared with the PBS- or GFP-dsRNA injected groups, and levels decreased by approximately 40% in the 80 µg-injected groups. With increasing injection doses of dsRNA (from 40 µg to 80 µg), the expression levels of the eight genes decreased. There was one exception, because the expression level of the 40 µg KRMP dsRNA-injected group remained at 95% after 6 days, but with 160 µg injection doses the expression level of KRMP was reduced by about 35% compared with the PBS or GFP dsRNA-injected group (Figure 5). We tested the expression of six known biomineralization-related genes (Pif, pearlin, and N19 involved in nacreous layer formation; prisilkin-39, aspein, and prismalin-14 involved in prismatic layer formation) as controls for the high dosage injection groups (160 µg dsRNA in the *krmp* knockdown group and 80 µg in the other gene knockdown groups). The relative mRNA levels of these six genes did not decrease or increase significantly, indicating that the RNAi strategy did not disrupt general gene expression in the epithelium (data not shown).

**Figure 6:**
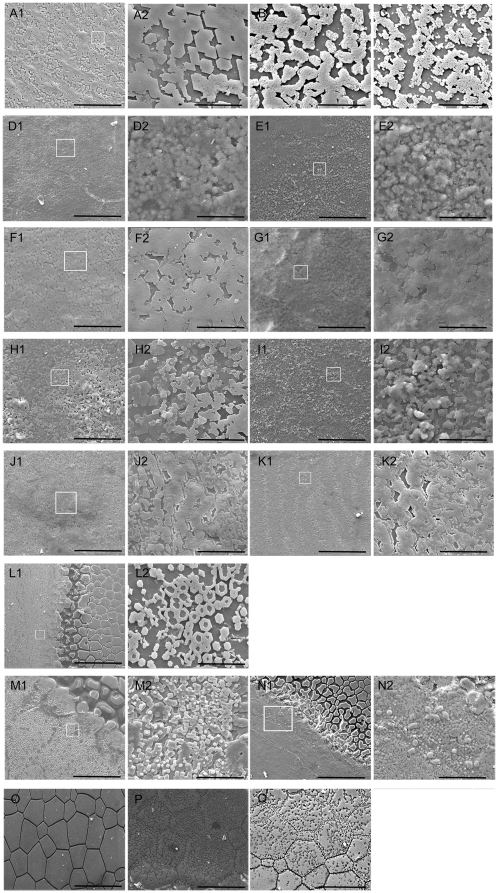
Effect of selected genes on shell growth. Expression levels of the selected genes were decreased by RNAi (see Materials and Methods for SEM image details). (A1) SEM image of the internal nacreous layer surface of the normal shell, showing stair-like growth pattern. (B–K2) SEM images of the internal nacreous layer surface of the shells in the dsRNA injected groups, which shows that the growth of the tablets was disrupted. (L1) SEM image of the ‘aragonitic line’ shows the growth of nacreous tablets on the left side and the growth of calcitic prisms on the right side. (M1–N2) SEM image of the ‘aragonitic line’ of the UD_Cluster94.seq.Singlet1-injected group. (O) SEM image of the normal prismatic layer of the shell. (P–Q) SEM image of the *krmp*-injected groups. Low dosage (80 µg for *krmp*, 40 µg for the other genes) injection of nacrein (B), DT_Cluster236 (D1), DT_Cluster252 (F1), DT_Cluster524.seq.Contig1 (H1), IU_Cluster32 (J1) UD_Cluster94.seq.Singlet1 (M1), and *krmp* (P). High dosage (160 µg for *krmp*, 80 µg for the other genes) injection of nacrein (C), DT_Cluster236 (E1), DT_Cluster252 (G1), DT_Cluster524.seq.Contig1 (I1), IU_Cluster32 (K1), UD_Cluster94.seq.Singlet1 (N1), and *krmp* (O). (A2, D2, E2, F2, G2, H2, I2, J2, K2, L2, M2, and N2) enlargement of the box in (A1, D1, E1, F1, G1, H1, I1, J1, K1, L1, M1, and N1). Bar  = 10 µm in A2, B, C, F2, G2, H2, J2, K2, L2, D2, E2, I2, M2; Bar  = 50 µm in A1, F1, G1, H1, J1, N2, P, Q; Bar  = 100 µm in D1, E1, I1, K1, M1, O, L1, N1.

To further investigate the role of these genes in shell biomineralization, we observed the inner surface structure of the shells six days after injection, *i.e*., internal nacreous layer, prismatic layer, and the transition section ‘aragonitic line’ that connect the nacreous layer and prismatic layer [24]).Compared with the untreated groups, the surfaces of shells in the PBS- and GFP dsRNA-injected groups had the same normal well-defined type of microstructure. Small rectangular or hexagonal flat tablets of aragonite were packed together in the nacreous layer to produce a stair-like growth pattern (Figures 6A1, 6A2). The ‘aragonitic line’ contained flat tablets of aragonite on one side and calcitic prisms on the other side (Figures 6L1, 6L2). Prisms built up into a well-compacted smooth structure in the prismatic layer (Figure 6O).

Surfaces of the internal nacreous layers were disordered in the five injected groups, *i.e*., nacrein, DT_Cluster236, DT_Cluster252, DT_Cluster524.seq.Contig1, and IU_Cluster32. The normal structures were disturbed, either by random accumulation of crystals (DT_Cluster236, DT_Cluster252, and DT_Cluster524.seq.Contig1, Figures 6 D1–I2), or by changing the shape of tablets (nacrein and IU_Cluster32, Figures 6 B, C, J1–K2). These abnormal characteristics were more significant in the high dosage (80 µg) injected groups compared with the low dosage (40 µg) injected groups (Figures 6B–K2). Surfaces of the prismatic layers and the ‘aragonitic line’ were normal and ordered like the untreated control. These results suggest that the four newly-identified genes participate with nacrein in the formation of the internal nacreous layer.

Some cube-shaped tablets appeared at the nacreous side of the ‘aragonitic line’, which disrupted shell structure in the low dosage (40 µg) UD_Cluster94.seq.Singlet1 dsRNA-injected group (Figures 6M1, M2). In the high dosage (80 µg) injected group, the edges of the cube-shaped tablets were obscured by the changed shape of the tablets and some different-shaped, bigger tablets were formed by the fusion of crystals (Figures 6N1, N2). The surfaces of the nacreous layer and prismatic layer were ordered like normal shells. These observations indicate that this gene can modify the shape of the tablets on the ‘aragonitic line’, but there were no effects on the other shell sections.

The KRMP expression level in the *krmp* 40 µg dsRNA-injected group did not decrease significantly and the shell surface remained normal, as expected. In the 80 µg injected groups, the calcitic prisms that formed the prismatic layer were abnormal. The surface of the calcitic prisms was lacunose and their borders were broken (Figure 6P). At a high dosage (160 µg) injection, we found similar changes in the calcitic prisms of the prismatic layer (Figure 6Q). Structures of the internal nacreous layer and the ‘aragonitic line’ were normal in all *krmp* dsRNA-injected groups. There were no obviously disordered structures in the prismatic layers of the other gene-injected groups.

RNAi showed that there was a possibility that disturbance of growth in both layers of the shell could lead to mantle secretion of proteins to overcome disordered growth in one layer. To confirm this, we injected 80 µg nacrein dsRNA and 80 µg *krmp* dsRNA together. The structures changed in both the nacreous layer and prismatic layer, and the abnormal characteristics seen in each layer matched those seen in the separately injected groups (Figure S3).

## Discussion

Three SSH libraries were constructed to isolate genes expressed at different developmental stages during new shell formation. Changes of crystals and the microstructure of the shell were correlated with biomineralization-related sequences in the D–T library that were involved in ACC formation, sequences in the U–D library were involved in aragonite formation, and sequences in the J–U library were involved in the formation of the microstructure of the nacreous and prismatic layers.

Bioinformatics analysis showed that only a small number of sequences in the three libraries were with associated GO terms. This was similar to other non-model invertebrate EST data sets [74], [75], [76], [77]. The main classes of annotated genes were the same in the three libraries and they closely matched the mantle transcriptome analysis of the tropical abalone *Haliotis asinine* and the silver lipped pearl oyster *Pinctada maxima*, where shared ESTs (removed genes encoding riboproteins, rRNAs, and mitochondrial proteins) were annotated with GO terms [78]. The cutoff e-value used in our annotation pipeline was 10e-05, which means sequences with low similarity would be excluded in the GO hits. Novel sequences and sequences with low similarity were not included in the GO annotation, but the GO annotation revealed that the main classes of sequences in the three libraries were putative genes expressed by the mantle that directly control shell biomineralization.

Careful analysis of BLASTX results found some sequences similar to known biomineralization-related genes (Table S1). In the D–T library, sequences were found with similarities to chitin synthase and chitin-binding protein. Chitin is an insoluble polysaccharide that forms the highly structured framework of the shell. In the shell formation of the marine bivalve mollusc *Mytilus galloprovincialis*, the chitinous material is present in the larval shell, presumably as a chitin–protein complex [48]. Chitin synthase fulfills an important enzymatic role in the coordinated formation of larval bivalve shells [46], so a similar process might be present during larval shell formation in *P.fucata*, where chitinous material was synthesized in the D-shape stage.

ACC is considered to be a precursor of crystalline aragonite or calcite, but it is also a component of the D-shape larval shell. ACCBP shares significant sequence similarity with a group of acetylcholine-binding proteins and controls the formation of ACC in *P.fucata*. It might inhibit the growth of calcite and induce the formation of ACC. This capacity of ACCBP is directly related to its acetylcholine-binding site. DT_Cluster236 shared similarity with nicotinic acetylcholine receptor in *Takifugu rubripes*, which indicates that it has an acetylcholine-binding site gene that might participate in the control of ACC in the D-shape larval shell formation. We investigated this function in later experiments. We found some sequences in the U–D library that were similar to calcium metabolism-associated proteins (CaM, CaM-KK, and BMP). This may be due to the fact that a large amount of calcium is required during umbo shell formation. MSI60 was involved in the nacreous layer during adult shell formation, and was present in the J–U library. This result agreed with previous work [36] that showed MSI60 expression was initiated in 31-day-old larvae. Lectin, fibrinogen, cysteine-rich protein, ferritin, MSI7, and collagen were previously found to be involved in biomineralization. We found unigenes in our libraries that had similarity with these biomineralization genes. These unigenes might participate in shell formation, and investigation of their structure-function relationships would enhance our understanding of biomineralization. Further studies of these sequences are ongoing.

We searched genes coding for secreted proteins and containing tandem-arranged repeat units to identify genes involved in shell formation. This strategy was used for screening genes involved in shell formation in *H.asinina* and *P.maxima* [78] and previous results suggested that these isolated genes are indeed critical to the process of shell formation. The four unigenes coding for secreted proteins with tandem-arranged repeat units in our three libraries were important candidate genes for direct involvement in shell formation. BLASTX searching found no significant similarity with sequences in the GenBank nr database. Distribution of the candidate unigenes in mantle tissue was analyzed so as to understand their functions. Mantle tissue is considered to directly control the formation of shell. The shell matrix protein genes had regional expression patterns along the mantle, and the distributions of the genes suggested their functions in shell biomineralization. Together with the ACC control-related sequence DT_Cluster236, we analyzed the spatial expression of the five genes in several target tissues, especially the mantle tissue. The results showed that they were all expressed in the mantle with different distributions from known biomineralization related genes. DT_Cluster236, DT_Cluster252 and IU_Cluster32, were expressed by the mantle and other tissues. These genes may be secreted into the body fluids after synthesis in mantle tissues and they might protect from abnormal crystallization in inappropriate locations, as does ACCBP. Prismatic layer genes were mostly expressed in the periostracal groove of the mantle, whereas DT_Cluster236, DT_Cluster252 and IU_Cluster32 were expressed in both mantle edge and mantle pallial indicating that these genes were not involved in the prismatic layer formation. This was supported by subsequent RNAi analysis.

To elucidate the role of these five genes in shell formation, dsRNAs were injected into the oyster to knock down expression levels. The expression levels of these genes decreased to 50∼60% in low dosage (40 µg or 80 µg for *krmp*) injected groups, and to 40% in high dosage (80 µg or 160 µg for *krmp*) injected groups (Figure 5). Together with the SEM images (Figure 6) of the inner surface of the shells, we found that with more dsRNA injected, the expression levels of genes decreased more and shell defects phenoma became increasingly noticeable. Compared with other genes, more *krmp* dsRNA was needed to effectively knock down KRMP expression. The KRMP family has four homologs in *P.fucata*. The dsRNA would affect the four genes, and expression level we analyzed by qPCR was that of the four genes. Thus, it is not surprising that more dsRNA was required. After *krmp* genes were knocked down, the prismatic layer showed defects on the border of calcitic prisms. The calcitic prisms formed a loosely organized structure. The abnormal shell structures may be caused by a poor connection between the prisms and the framework [20], [79].

Nacrein plays an important role as a synthesizer and carrier of calcium carbonate in aragonite crystallization [80]. However, later studies demonstrated that the specific involvement of nacrein in the nucleation of aragonite crystal was doubtful [81], [82], [83]. In the nacrein dsRNA injected groups, the shell defects only appeared in the nacreous layer, and the characteristic matched those seen in earlier nacrein antibody injection experiments [84]. Our nacrein RNAi study supported the former, but not the latter, hypothesis that nacrein was involved in nacreous layer formation. As expected, the five gene candidates affected the formation of the nacreous layer. The specific abnormal characteristics of the nacreous layer were not the same in the injected groups and they could be broadly classified into two types: random accumulation of crystals, as seen in the DT_Cluster236-, DT_Cluster252-, or DT_Cluster524.seq.Contig1-injected groups; and tablet shape changes, as seen in the other two unigene-injected groups. Two antagonistic mechanisms are involved in the control of shell formation: crystal nucleation and growth inhibition [27]. Nacrein, prisilkin-39, ACCBP and Pif are well-studied in the shell formation of *P.fucata*. By reviewing the shell defects caused by repression of them and their functions in the control of calcium carbonate crystals [16], [21], [33], [84], we found that repression of genes that induce the nucleation of aragonite crystals, *i.e*., nacrein and Pif, led to changes in aragonitic tablet shape, whereas repression of genes inducing aragonite crystal growth, *i.e*., prisilkin-39 and ACCBP, led to random accumulation of crystals. We hypothesized that the random accumulation of crystals was caused by defects in growth inhibition, and the change of tablet shape was caused by crystal nucleation defects.

It is very interesting that knock down of the UD_Cluster94.seq.Singlet1 affected the ‘aragonitic line’ formation. The ‘aragonitic line’ is the sharp separation between aragonite and calcite, where the longitudinal growth of calcitic prisms is prevented and new aragonitic crystals are formed. The mineralogy, shape, size, and growth modalities of biocrystals are completely changed. However, little is known about the ‘aragonitic line’, which is a very narrow section in the inner surface of the shell that is hard to separate it from the shell and it is very difficult to isolate proteins directly from this section. Our U–D library was constructed to isolate genes that participated in the initiation of nacreous formation, which also occurs at the ‘aragonitic line’. Thus it was not surprising that UD_Cluster94.seq.Singlet1 was involved in ‘aragonitic line’ formation. UD_Cluster94.seq.Singlet1 affected the inner surface of shell on the aragonitic side, where the aragonitic tablets were barely formed and inhibited the progress of crystal growth. The tablets were hexagonal, and some possessed a hollow structure. However, the mature aragonitic tablets in the inner surface of the internal nacreous layer were bigger and well compacted with no hollow structure. The UD_Cluster94.seq.Singlet1 did not affect the internal nacreous layer, but only the nacreous side of the ‘aragonitic line’. Thus, the control mechanism of aragonitic crystal initiation was different from mature aragonitic crystal growth. The aberrant textures seen in this injected groups were very similar to those seen in diseased specimens [24], which suggest that the pearl oyster may experience the same defective process under natural conditions. Further structure-function relationship studies of this gene are ongoing and we expect that this will bring us closer to an understanding of shell formation. Future efforts to use morpholino oligos to knock down gene expression in larvae would greatly help us understand the function of these genes and shell formedation.

There were two methods of investigation to find the mechanism of shell formation: one is to investigate the components, such as the proteins that participate in formation; the other one is to investigate the formation pathways. This study investigated the proteins involved in biomineralization, but the pathways are also very important in understanding the shell formation. We will identify the pathways in our subsequent work.

## Materials and Methods

### Ethics Statement

*N/A*

### Larval culture

Larval samples of *P. fucata* were collected from the Guofa Pearl Farm in Beihai, Guangxi Province, China. The insemination process was described in Fujimura *et al.* [34]. Fertilized eggs were incubated at 25°C. Different samples were obtained at 17 h, 22 h, 7 days, or 35 days after fertilization, respectively.

### RNA extraction and cDNA synthesis

Total RNA was extracted from the larval stages using the SV total RNA isolation kit (Promega), according to the manufacturer's instruction. RNA quantity was assessed by measuring OD_260/280_ with an Utrospec 3000 UV-visible spectrophotometer (Amersham Biosciences). The integrity of RNA was determined by fractionation on 1.2% formaldehyde denatured agarose gel and staining with ethidium bromide. In the subtractive hybridization procedure, 0.5 µg of total RNA was used to produce double strand cDNA using the SMART™PCR cDNA synthesis kit (Clontech). We used 1 µg of total RNA as the template for reverse transcription to synthesize first-strand cDNA by MMLV-RT reverse transcriptase (Promega) and oligo(dT) (Promega) as the primer.

### Suppression subtractive hybridization and library construction

We performed suppression subtractive hybridization procedures with different development stages. The D-T library was constructed using cDNA prepared from early D-shape stage (20-hour-old larva) as the tester and the trochophore stage (14-h-old larva) as the driver. The U-D library used cDNA prepared from the umbonal stage (7-day-old larva) as the tester and that from D-shape stage as the driver. And the J–U library was obtained using cDNA from the juvenile stage (35-day-old larva) and umbonal stage as tester and driver.

We applied SSH using a PCR-Select™cDNA Subtraction kit (Clontech), according to the manufacturer's instructions. To evaluate subtraction efficiency, the abundance of transcripts of the housekeeping gene *gapdh* was examined by PCR using primer pairs of *gapdhF* and *gapdhR* (see Data S1 for primer details). The PCR-amplified subtracted cDNA was inserted into the T/A cloning vector pGEM-T easy vector (Promega) and transformed into Trans2-Blue Chemically Competent Cell (TransGen), following the manufacturer's instructions.

To evaluate whether inserts were the enriched cDNA of the tester, 3,515 clones from the three libraries were PCR amplified using paired primers (Nested Primer1 and Nested Primer 2R) with the PCR-Select™cDNA Subtraction kit (Clontech), then analyzed by electrophoresis on a 1.2% agarose gel to estimate the size of inserts. The PCR products were combined with an equal volume of 0.6 N NaOH and manually spotted onto a nylon membrane. The membrane was hybridized with DIG-labeled cDNA probes synthesized for the tester and driver. The hybridized membrane was exposed to X-ray film to show the positive clones. This was conducted according to the manufacturer's instructions with the DIG High Prime DNA labeling and detecting starter kit II (Roche Applied Science). Finally, 2,975 clones with positive signals in tester cDNA hybridization and negative signals in driver cDNA hybridization were selected from the three libraries. Raw sequences were submitted to the NCBI dbEST database (GenBank: GW602961– GW603856).

### Sequence analysis and bioinformatics

We selected 2,975 positive clones for sequencing and 2,923 available sequences were returned. These sequences were edited and assembled using the programs PHRED, CROSS_MATCH, and CONSED (http://bozeman.mbt.washington.edu/). Vector sequences were removed and the remaining sequences with a minimum size of 100 bases were taken for further analysis. Remaining sequences were clustered using the TGICL [85] and assembled into 990 unigenes. BLASTX search with the GenBank nr database was used to annotate sequences, and an e-value of 10e–05 was used to cutoff annotations with low similarities. Most of the sequences returned no hits, so we used Blast2GO [42] for gene ontology (GO) assignment. The GO annotation results were classified using WEGO [43]. Unigenes were searched against the transcriptome of the mantle tissue by BLASTN annotation, with a cutoff e-value of 10e–06. Coding regions were identified by aligning the unigenes by BLASTN (cutoff e-value 10e–05) to protein databases in the priority order of GenBank nr, Swiss-Prot, KEGG, and COG. Unigenes aligned to databases with higher priority did not proceed to the next step. The alignments ended when all circles were finished. Proteins with highest ranks in BLAST results were used to determine the coding region sequences of snigenes and coding region sequences were translated into amino acid sequences. Thus, the nucleotide sequences (5′–3′) and amino sequences of the unigene coding regions were acquired. Unigenes that could not be aligned to any database were investigated by ESTScan [86] of nucleotide sequences (5′–3′) and amino sequences of the coding regions.

In secreted protein research, the unigenes with a coding region shorter than 150 base pairs and with no stop codon were discarded. The remaining potential full-length coding regions were searched for signal peptides using SignalP v3.0 [87]. Signal peptide-positive unigenes were searched using TargetP [88] to remove proteins targeted to organelles. Transmembrane proteins were removed using the TMHMM Server v. 2.0 (http://www.cbs.dtu.dk/services/TMHMM/) to predict transmembrane domains. Glycosyl phosphatidyl inositol-anchored proteins were discarded using the GPI modification predictor [89]. The remaining unigenes were considered to code for secreted proteins [90]. We also searched for known matrix proteins in *P.fucata* as a control.

We used XSTREAM [73] to isolate genes coding for proteins with tandem-arranged repeat units. In our search, unigenes with a coding region shorter than 150 base pairs were discarded. The settings used in this analysis were as the default, *i.e*., degeneracy  = 0, TR significance  =  high, and min consensus match  = 0.8. The known matrix proteins of *P.fucata* were included as the control.

### Gene expression analysis by RT-PCR assay

Tissue expression in the pearl oyster was investigated by RT-PCR. Total RNA was isolated using the method described above for mantle edge, mantle pallial, gill, adductor muscle, gonad, foot, hemocytes, and viscus of adult individual *P. fucata*. Equal quantities (1 µg) of total RNA from different tissues were reverse-transcribed into the cDNA first strand using Quant Reverse Transcriptase (Tiangen), following the manufacturer's instructions. The synthesized cDNA was used as a template for the following PCR. GAPDH was used as a positive control for cDNA preparations (see Data S1 for primers details). Negative controls were conducted in the absence of the cDNA template to test for cross-contamination of the samples. All PCR products were subcloned and verified by sequencing.

### *In situ* hybridization

Adult *P.fucata* mantle was removed and immediately fixed overnight in 4% paraformaldehyde containing 0.1% diethyl pyrocarbonate (Sigma). *In situ* hybridization was performed on frozen mantle sections. The fragment was amplified with the primer pair of the genes (see Data S1 for details) and inserted in multiple cloning sites of the pGEM-T Easy Vector (Promega) and digoxigenin-labeled RNA probes were generated using a DIG RNA Labeling Kit (Roche). *In situ* hybridization was performed as described previously [91]. To avoid false positive signals, the hybridization temperature was increased to 50°C.

### RNAi experiments

RNAi was performed as described in Suzuki *et al*. [16], with some modifications. Different primers (see Data S1 for details) were used to amplify specific sequences from the first-strand cDNA for GFP, while pEGFP-C1 (Clontech) was used as the template. The PCR products were purified using Wizard PCRPrepDNA purification system (Promega). A RiboMAX™ Large Scale RNA Production Systems (T7) kit (Promega) was used to synthesize and purify the dsRNA. RNase free DNase I (TAKARA) was used to digest the template DNA. The integrity and quantity of the dsRNA were tested as previously described. The dsRNA were diluted to 40, 80, or 160 µg 100 µL^–1^ with PBS, and 100 µL solutions were injected into the adductor muscle of 2-year-old individuals with a shell length of 5–6 cm. 100 µL PBS and 40 µg GFP dsRNA in 100 µL PBS were used as controls. Five individuals were used in each treatment.

We extracted total RNA from the mantle tissue of each oyster 6 days after injection and synthesized first-strand cDNA, as described above. Real-time quantitative PCR (qPCR) was used to quantity the expression levels of each gene, and β-actin was used as an internal reference. qPCR was conducted with the Mx3000PTM (Stratagene) using an SYBR® Premix Ex Taq™II kit (TAKARA), according to the manufacturer's instructions (see Data S1 for primer details.) Cycling parameters were: 95°C for 30 s (1 cycle); 95°C for 5 s; 55°C for 30 s; 72°C for 30 s (40 cycles). Dissociation curves were analyzed to determine the purity of the product and specificity of amplification.

Six days after injection, the shells of the injection groups were thoroughly washed with Mili-Q water and air-dried. Shells were cut into pieces that mounted on the scanner with the inner nacreous surface face-up, sputter-coated with 10-nm-thick gold, and analyzed using an FEI Quanta 200 scanning electron microscope. Areas at the centre and edge of the shell were examined as the internal nacreous layer. Cross-sectional views were obtained by viewing from the edge of the shell.

## Supporting Information

Figure S1 **Verification of SSH procedure.** Test of the reduction of GAPDH abundance. PCR was performed on the subtracted (Lanes 1–4) or unsubtracted (Lanes 5–8) secondary PCR product using the GAPDH 5′ and 3′ primers. Lanes 1, 5: 18 cycles; Lanes 2, 6: 23 cycles; Lanes 3, 7: 28 cycles; Lanes 4, 8: 33 cycles. Lane M: marker. (a) Analysis of genes in the D–T library. (b) Analysis of genes in the U–D library. (c) Analysis of genes in the J–U library. The GAPDH abundance decreased by a factor of at leats 1∶32 in the subtracted library compared with the unsubtracted samples.
(PDF)

Figure S2 **GO annotations for unigenes in the three SSH libraries.** The unigenes were first annotated by BLASTX using the Genbank nr database with a cutoff e-value of 10e–05. Blast2GO [42] was used for gene ontology (GO) assignment. The GO annotation results were classified using WEGO [43]. The white columns represent unigenes in the D–T library, the grey columns represent unigenes in the U–D library, and dark grey columns represent unigenes in the J–U library.
(PDF)

Figure S3 **Knockdown of the *krmp* and nacrein genes by RNAi.** (A) The gene expression level of the genes six days after 80 µg nacrein dsRNA and 80 µg *krmp* dsRNA injection. (B) SEM images of the inner surface of the shell. a, SEM image of internal nacreous layer showing that nacreous tablets were disrupted. b, SEM image of prismatic layer. The border of the calcitic prisms appears to be broken. c, SEM image of the ‘aragonitic line’. The microstructure of this section was normal. d. enlargement of the box in c. Bar  = 10 µm in a, b, d. Bar  = 50 µm in c.
(PDF)

Table S1 **Unigenes from the three SSH libraries with homology to biomineralization-related genes.**
(PDF)

Data S1 **Primers used for the test of the reduction of GAPDH abundance, *in situ* hybridization probe synthesis, dsRNA synthesis and real-time quantitative PCR.**
(PDF)
