# Supplementary figures and images for: Identification of Genes Directly Involved in Shell Formation and Their Functions in Pearl Oyster, Pinctada fucata
Source: PLoS One. 2011 Jul 1;6(7):e21860. doi: 10.1371/journal.pone.0021860 (PMC3128620; doi:10.1371/journal.pone.0021860)

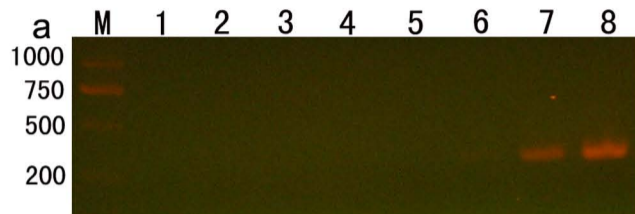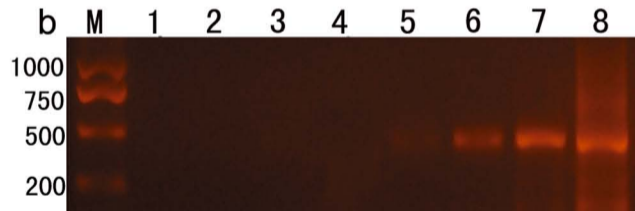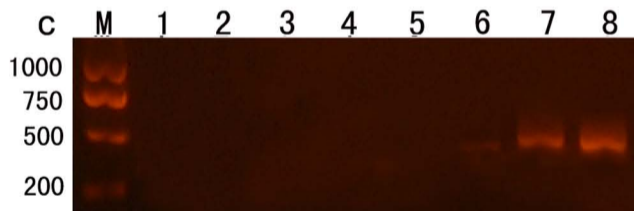

Supplement: Figure S1 — Verification of SSH procedure. Test of the reduction of GAPDH abundance. PCR was performed on the subtracted (Lanes 1–4) or unsubtracted (Lanes 5–8) secondary PCR product using the GAPDH 5′ and 3′ primers. Lanes 1, 5: 18 cycles; Lanes 2, 6: 23 cycles; Lanes 3, 7: 28 cycles; Lanes 4, 8: 33 cycles. Lane M: marker. (a) Analysis of genes in the D–T library. (b) Analysis of genes in the U–D library. (c) Analysis of genes in the J–U library. The GAPDH abundance decreased by a factor of at leats 1∶32 in the subtracted library compared with the unsubtracted samples. (PDF) [file pone.0021860.s001.pdf]

# GO-Standard

D-T  
 U-D  
 J-U

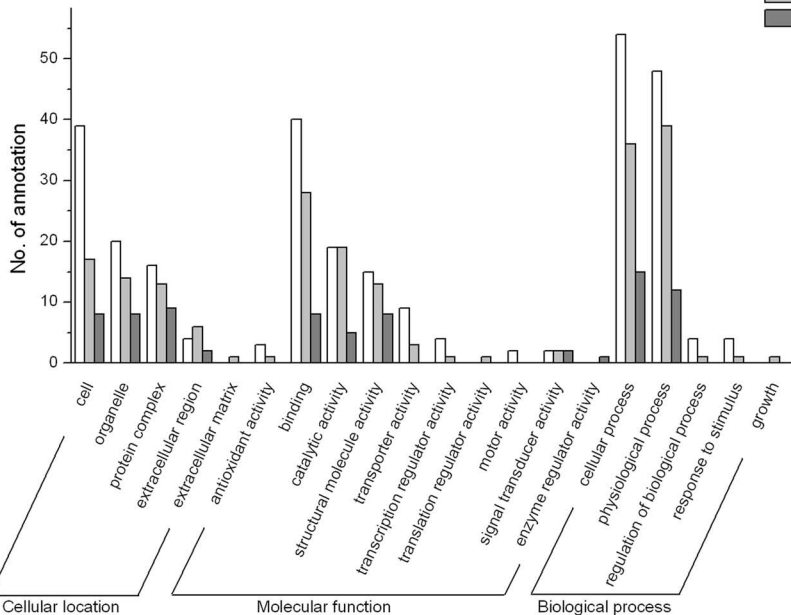

Supplement: Figure S2 — GO annotations for unigenes in the three SSH libraries. The unigenes were first annotated by BLASTX using the Genbank nr database with a cutoff e-value of 10e–05. Blast2GO [42] was used for gene ontology (GO) assignment. The GO annotation results were classified using WEGO [43]. The white columns represent unigenes in the D–T library, the grey columns represent unigenes in the U–D library, and dark grey columns represent unigenes in the J–U library. (PDF) [file pone.0021860.s002.pdf]

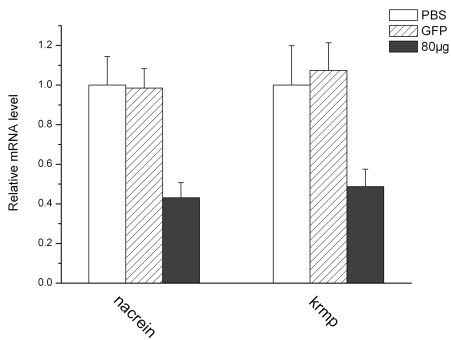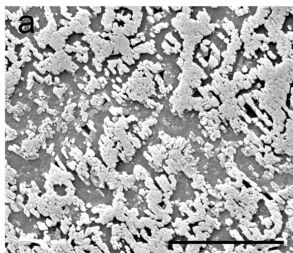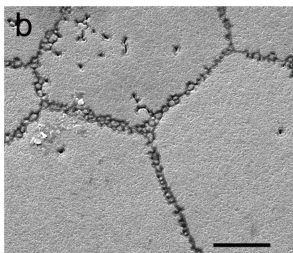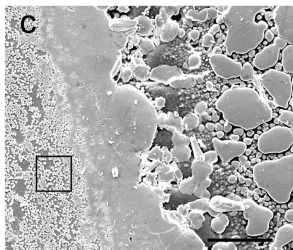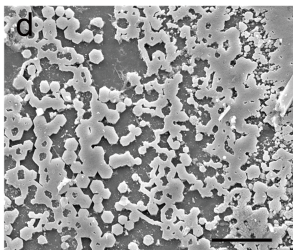

Supplement: Figure S3 — Knockdown of the krmp and nacrein genes by RNAi. (A) The gene expression level of the genes six days after 80 µg nacrein dsRNA and 80 µg krmp dsRNA injection. (B) SEM images of the inner surface of the shell. a, SEM image of internal nacreous layer showing that nacreous tablets were disrupted. b, SEM image of prismatic layer. The border of the calcitic prisms appears to be broken. c, SEM image of the ‘aragonitic line’. The microstructure of this section was normal. d. enlargement of the box in c. Bar = 10 µm in a, b, d. Bar = 50 µm in c. (PDF) [file pone.0021860.s003.pdf]
