# Supplementary material for: Identification of Genes Directly Involved in Shell Formation and Their Functions in Pearl Oyster, Pinctada fucata
Source: PLoS One. 2011 Jul 1;6(7):e21860. doi: 10.1371/journal.pone.0021860 (PMC3128620; doi:10.1371/journal.pone.0021860)
Supplement: Table S1 — Unigenes from the three SSH libraries with homology to biomineralization-related genes. (PDF) [file pone.0021860.s004.pdf]

Table S1. Unigenes from the 3 SSH libraries with homology to biomineralization related genes.

Table S1. A. The unigenes with homology to biomineralization related genes in the D-T library.

Unigene name	cDNA length (bp)	Homologous genes No.	E-value	Homologous genes
DT_Cluster10	281	gi|157114029	3.00E-16	**Fibrinogen and Fibronectin** [*Aedes aegypti]*
DT_Cluster160	540	gi|152205944	2.00E-15	**Chitin synthase** [*Pinctada fucata*]
DT_Cluster236	154	gi|31790121	2.00E-10	**Nicotinic acetylcholine receptor** alpha 9d subunit [*Takifugu rubripes*]
DT_Cluster279<br>DT_Cluster527.seq.Contig1	286<br>344	gi|241726688	1.00E-14	peritrophic membrane **Chitin Binding Protein** putative [*Ixodes scapularis*]
DT_Cluster281	269	gi|241818165	3E-16	**cysteine-rich** motor neuron, putative [*Ixodes scapularis*]
DT_Cluster32<br>DT_Cluster512.seq.Contig1	320	gi|194669778	8E-13	**Collagen, type XII,** alpha 1 isoform 1 [*Bos taurus]*
DT_Cluster520.seq.Contig1	716	gi|198420236	3.00E-16	**Collagen type VI** alpha 6 [*Ciona intestinalis*]
DT_Cluster343	293	gi|193999217	2.00E-47	**Heat shock protein 70** [*Pinctada fucata*]
DT_Cluster398	261	gi|93139010	4.00E-12	**Ferritin** [*Crassostrea ariakensis*]
DT_Cluster447	307	gi|126697410	2.00E-26	sialic acid binding **lectin** [*Haliotis discus discus*]
DT_Cluster550.seq.Contig1	291	gi|154816107	7.00E-28	C-type **lectin**-1 [*Crassostrea gigas*]

Table S1. B. The unigenes with homology to biomineralization related genes in the U-D library.

Unigene name	cDNA length (bp)	Homologous genes No.	E-value	Homologous genes
UD_Cluster193. seq.Contig1	476	gi|32563747	2.00E-06	**Collagen family member** (col-17) [*Caenorhabditis elegans*]
UD_Cluster81. seq.Singlet1	466	gi|4519619	4.00E-09	**Collagen** pro alpha-chain [*Haliotis discus*]
UD_Cluster67. seq.Singlet1	291	gi|154816107	1.00E-27	C-type **lectin**-1 [*Crassostrea gigas*]
UD_Cluster109. seq.Singlet1	324	gi|33333949	3.00E-50	**Ferritin-like protein** [*Pinctada fucata*]
UD_Cluster121. seq.Singlet1	461	gi|156399584	1E-40	**Calmodulin** [*Metridium senile*]
UD_Cluster160. seq.Contig1	216	gi|126697410	3.00E-14	sialic acid binding **lectin** [*Haliotis discus discus*]
UD_Cluster167. seq.Singlet1	374	gi|194217502	2.00E-06	**calcium/calmodulin-dependent protein kinase kinase 1, alpha** [*Equus caballus*]
UD_Cluster187. seq.Contig1	300	gi|33325246	5.00E-06	glycine-rich shell matrix protein (**MSI7**) [*Pinctada fucata*]
UD_Cluster80. seq.Singlet1	472	gi|196049300	5.00E-49	**BMP5-8** [*Saccostrea kegaki*]

Table S1. C. The unigenes with homology to biomineralization related genes in the J-U library.

Unigene name	cDNA length (bp)	Homologous genes No.	E-value	Homologous genes
IU_Cluster13	216	gi|126697410	3E-14	**sialic acid binding lectin** [*Haliotis discus discus*]
IU_Cluster16	228	gi|93139010	2.00E-06	**ferritin** [*Crassostrea ariakensis*]
IU_Cluster36	269	gi|94471616	9.00E-41	soma **ferritin** [*Aplysia californica*]
IU_Cluster64. seq.Contig1	219	gi|33325246	1E-110	glycine-rich shell matrix protein **(MSI7**) [*Pinctada fucata*]
IU_Cluster7	218	gi|2204080	1.00E-111	mRNA for insoluble protein (**MSI60**) [*Pinctada fucata*]
