# Supplementary material for: Identification of Genes Directly Involved in Shell Formation and Their Functions in Pearl Oyster, Pinctada fucata
Source: PLoS One. 2011 Jul 1;6(7):e21860. doi: 10.1371/journal.pone.0021860 (PMC3128620; doi:10.1371/journal.pone.0021860)
Supplement: Data S1 — Primers used for the test of the reduction of GAPDH abundance, in situ hybridization probe synthesis, dsRNA synthesis and real-time quantitative PCR. (PDF) [file pone.0021860.s005.pdf]

Data S1. Primers used for the test of the reduction of GAPDH abundance, in situ hybridization probe synthesis, double strain RNA synthesis and real-time quantitative PCR.

A. Primers used for the test of the reduction of GAPDH abundance

GAPDH:

Forward: gapdhF: 5’-TCTGCTGATGCTCCTATGTTTG-3’
Reverse: gapdhR: 5’-TCTGGTATAACTTTGCCTACGG-3’

B. Primers used for the in situ hybridization probe synthesis and RT-PCR

GAPDH:

ingapdhF: 5’-TCTGCTGATGCTCCTATGTTTG-3’
ingapdhR: 5’-TCTGGTATAACTTTGCCTACGG-3’

DT_Cluster236:

inD1F: 5’- GATTCAGGGGGAAGCAC- 3’
inD1R: 5’-ATACGACGCAAAGCCAC-3’

DT_Cluster252:

inD2F: 5’-CGTCCCAGCCCTACTTTTTA-3’
inD2R: 5’-TGACCCTCATTGGATGTTCT-3’

DT_Cluster524.seq.Contig1:

inD3F: 5’-TGTCCAGTATAAGTCTGTCGA-3’
inD3R: 5’-TTAAAAGGTCAAAGGTCAAG-3’

UD_Cluster94.seq.Singlet1:

inUF: 5’-TTGTGAGTTGAACTGGTCGGGATC-3’
inUR: 5’-GAAGCAGTGGCAGCGGAAGC-3’

IU_Cluster32:

inIF: 5’-TGACCCTCGCACTGGTATTG-3’
inIR: 5’-GATTGGCTCTGATTCGTCCT-3’

C. Primers used for the double strand RNA synthesis, the underlined sequences are T7 promoters.

DT_Cluster236:

dsD1F: 5’- <u>GCGTAATACGACTCACTATAGGGAGA</u>GATTCAGGGGGAAGCAC- 3’
dsD1R: 5’-<u>GCGTAATACGACTCACTATAGGGAGA</u>ATACGACGCAAAGCCAC-3’

DT_Cluster252:

dsD2F: 5’-<u>GCGTAATACGACTCACTATAGGGAGA</u>CGTCCCAGCCCTACTTTTTA-3’
dsD2R: 5’-<u>GCGTAATACGACTCACTATAGGGAGA</u>TGACCCTCATTGGATGTTCT-3’

DT_Cluster524.seq.Contig1:

dsD3F: 5’-GCGTAATACGACTCACTATAGGGAGATGTCCAGTATAAGTCTGTCGA-3’
dsD3R: 5’-GCGTAATACGACTCACTATAGGGAGATTAAAAGGTCAAAGGTCAAG-3’

UD_Cluster94.seq.Singlet1:
dsUF: 5’-GCGTAATACGACTCACTATAGGGAGATTGTGAGTTGAACTGGTCGGGATC-3’
dsUR: 5’-GCGTAATACGACTCACTATAGGGAGAGAAGCAGTGGCAGCGGAAGC-3’

IU_Cluster32:
dsIF: 5’-GCGTAATACGACTCACTATAGGGAGATGACCCTCGCACTGGTATTG-3’
dsIR: 5’-GCGTAATACGACTCACTATAGGGAGAGATTGGCTCTGATTCGTCCT-3’

krmp:
dsKF: 5’-GCGTAATACGACTCACTATAGGGAGAAAGTTCGCCGCTGTTTTGGC-3’
dsKR: 5’-GCGTAATACGACTCACTATAGGGAGACATATCCACCACTGGATCCTGG-3’
nacrein:
dsNF: 5’-GCGTAATACGACTCACTATAGGGAGAGGCTTTGGCGACGAACCGGA-3’
dsNR: 5’-GCGTAATACGACTCACTATAGGGAGAACACGGGGGAGTGGTCAGGG-3’

GFP:
dsEF:5’- GCGTAATACGACTCACTATAGGGAGAATGGTGAGCAAGGGCGAGGAG-3’
dsER: 5’-GCGTAATACGACTCACTATAGGGAGATTACTTGTACAGCTCGTCCATG-3’

D. Primers used in the real-time quantitative PCR

qPCR primers were designed using the BeaconDesigner7.7 software (PREMIER Biosoft International).

DT_Cluster236:
qD1F: 5’-GAAGCACGAATCCTAGAG-3’
qD1R:5’- TTCACATACGACGCAAAG-3’

DT_Cluster252:
qD2F: 5’-TCCGCAATATAATGAACAAGT-3’
qD2R: 5’-TAGTAGACGCCACAATGAA-3’

DT_Cluster524.seq.Contig1:
qD3F: 5’-GTTTGTCTGTTTGTCTGTCT-3’
qD3R: 5’-CCCTATCACTTCCCATTCA-3’

UD_Cluster94.seq.Singlet1:
qUF: 5’-AGGATGATGGATCAAGCAT-3’
qUR: 5’-GTGGAGGAAGTAGCAGTAA-3’

IU_Cluster32:
qIF: 5’-TAAAGGTCATAGGTCAAAGGT-3’
qIR: 5’-GCTCTGATTCGTCCTCTTA-3’

krmp:
qKF: 5’-AAGAAATGTCACCCTTGGGATTGG-3’
qKR: 5’-AATCATCGCCACCATATCCATCG-3’

nacrein:
qNF: 5’-GGCTTTGGCGACGAACCGGA-3’
qNR: 5’-ACACGGGGGAGTGGTCAGGG-3’

pif:
qPifF: 5’-TGCTGCCATCACGTGAGTATG-3’
qPifR: 5’-GACTTCCCTTTCTCACACTTCCA-3’

pearlin:
qPeF: 5’-TACTCATACTGCTGGATA-3’
qPeR: 5’-TATCATCATCGGTGTAAC-3’

n19:
qN19F: 5’-CATCAACAAGTTCTCATTACAG-3’
qN19R: 5’-ATCTCCTACGGCTTACAG-3’

prisilkin-39:
qP39F: 5’-CTGGAATGAGAGGATATG-3’
qP39R: 5’-TGCTGCTGTAATAACTATA-3’

aspein:
qAF: 5’-TGATAGTGAAGACGATGA-3’
qAR: 5’-RTGTCATCATCATCATCATC-3’

prismalin-14:
qP14F: 5’-TAAGACCATTCTACGGCTAT-3’
qP14R: 5’-ACCATATCCTCCATATAATCCT-3’

β-actin:
qβ-actin-f: 5’-CTCCTCACTGAAGCCCCCCTCA-3’
qβ-actin-r: 5’-ATGGCTGGAATAGGGATTCTGG-3’
